# The Micellization of Well-Defined Single Graft Copolymers in Block Copolymer/Homopolymer Blends

**DOI:** 10.3390/polym13050833

**Published:** 2021-03-09

**Authors:** Eleni Pavlopoulou, Kiriaki Chrissopoulou, Stergios Pispas, Nikos Hadjichristidis, Spiros H. Anastasiadis

**Affiliations:** 1 Institute of Electronic Structure and Laser, Foundation for Research and Technology—Hellas, P.O. Box 1527, 71110 Heraklion Crete, Greece; kiki@iesl.forth.gr; 2 Theoretical and Physical Chemistry Institute, National Hellenic Research Foundation, 48 Vassileos Constantinou Ave., 11635 Athens, Greece; pispas@eie.gr; 3 Department of Chemistry, University of Athens, 15771 Athens, Greece; nikolaos.hadjichristidis@kaust.edu.sa; 4 Polymer Synthesis Laboratory, Physical Sciences and Engineering Division, King Abdullah University of Science and Technology (KAUST), Thuwal 23955, Saudi Arabia; 5 Department of Chemistry, University of Crete, 71003 Heraklion Crete, Greece

**Keywords:** block copolymers, micellization, single graft copolymers, small angle X-ray scattering, self-assembly

## Abstract

A series of well-defined (polyisoprene)_2_(polystyrene), I_2_S, single graft copolymers with similar total molecular weights but different compositions, *f_PS_*, were blended with a low molecular weight polyisoprene homopolymer matrix at a constant concentration 2 wt%, and the micellar characteristics were studied by small-angle x-ray scattering. To investigate the effect of macromolecular architecture on the formation and characteristics of micelles, the results on the single graft copolymers were compared with those of the corresponding linear polystyrene-*b*-polyisoprene diblock copolymers, SI. The comparison reveals that the polystyrene core chains are more stretched in the case of graft copolymer micelles. Stretching turned out to be purely a result of the architecture due to the second polyisoprene block in the corona. The micellization of a (polystyrene)_2_(polyisoprene), S_2_I, graft copolymer was also studied, and the comparison with the results of the corresponding I_2_S and SI copolymers emphasizes the need for a critical core volume rather than a critical length of the core-forming block, in order to have stable micelles. Finally, the absence of micellization in the case of the I_2_S copolymer with the highest polystyrene volume fraction is discussed. For this sample, macrophase separation occurs, with polyisoprene cylinders formed in the copolymer-rich domains of the phase-separated blends.

## 1. Introduction

The use of wisely chosen block or graft copolymers as compatibilizers in polymer blends is a well-established way to control interfacial adhesion between immiscible polymers and, thus, obtain an optimized product [1,2,3,4,5]. Block copolymers tend to preferentially reside at the interface between the two immiscible homopolymers [6,7,8,9,10,11] with each block preferentially extending into its corresponding homopolymer phase [7,11,12,13,14], and, thus, the interfacial tension between the phase-separated homopolymers [15,16,17,18,19] is reduced, and the interfacial adhesion [20,21,22] is enhanced. The efficiency of interfacial partitioning is predicted to depend on the molecular weights of the copolymer blocks relative to those of the homopolymers [10,12,23,24,25], on the macromolecular architecture/topology and composition of the copolymers [10,26,27,28,29,30,31,32,33,34,35,36,37,38], and on the interaction parameter balance between the homopolymers and the copolymer blocks [39,40]. 

However, the efficiency of the interfacial partitioning of diblock copolymers to the polymer/polymer interface can be severely affected by the formation of micelles within the homopolymer phases [10,23,24,25,41,42,43,44]. The micelles will compete with the interfacial region for copolymer chains, while the amount of copolymer at the interface or in micelles would depend on the relative reduction of the free energy of the system. We had previously investigated the effects of the block copolymer molecular weight, composition, and macromolecular architecture on the reduction of the interfacial tension between two immiscible homopolymers [42,45], where the results obtained were understood within a simple model that considered that interplay. The model considers the possibility of micelles formation as the additive molecular weight increases or as the copolymer composition or architecture is altered, which leads to a three-state equilibrium among copolymer chains adsorbed at the interface, chains homogeneously mixed with the bulk homopolymers, and copolymer chains participating in micelles within the homopolymer bulk phases. It is thus essential to understand the micellization behavior of copolymers of varying molecular weight, composition, and macromolecular architectures within homopolymer matrices.

The micellization of linear diblock copolymers added to a homopolymer matrix, which acts as a selective macromolecular solvent for one of the blocks, has been examined extensively both theoretically [12,46,47,48] and experimentally [49,50,51,52,53,54,55,56,57,58,59,60]. However, no reports appear in the literature concerning the micellization of non-linear copolymers within homopolymer matrices, apart from a few that deal with the formation of micelles within low molecular weight selective solvents [61,62,63,64,65,66,67,68,69,70,71,72,73,74]. 3-miktoarm (polystyrene)(polyisoprene)_2_, PS(PI)_2_, and (polystyrene)_2_(polyisoprene), (PS)_2_PI, star copolymers of similar molecular weights and compositions were studied [61] in *n*-decane, which is a selective solvent for the PI block, and the behavior was compared to that of the corresponding diblocks. 4-miktoarm star copolymers (polystyrene)(polyisoprene)_3_, PS(PI)_3_, of various molecular weights and compositions were investigated [62] in solvents selective for either one of the polymer blocks. Moreover, 12-miktoarm star copolymers (polystyrene)_6_/[poly(2-vinyl pyridine)]_6_, (PS)_6_(P2VP)_6_, were studied [63] in toluene, which is a selective solvent for the PS, as well as a 16-miktoarm star copolymer (polyisoprene)_8_(polystyrene)_8_, (PS)_8_(PI)_8_, in *n*-decane [64]. Star block copolymers of the type (polyisoprene-b-polystyrene)_8_, (PS-b-PI)_8_, with PI inner blocks were studied in two solvents selective for PS [65]. The micellar behavior of copolymers of even more complicated macromolecular architectures in low molecular weight solvents has also been investigated. Examples include the behavior of model super-H-shaped block copolymers (PI)_3_(PS)(PI)_3_, which were investigated in *n*-decane by small-angle neutron scattering, light scattering, and viscometry [66], as well as the behavior of model graft copolymers of the H-type, (PS)_2_(PI)(PS)_2_, and of the π-type, (PS,PI)PI(PI,PS), in solvents selective for the PI backbones or the PS branches [75]. Wherever possible, the results were compared with those obtained from the respective linear diblock copolymers, with block lengths equal to the lengths of the corresponding arms, in order to isolate the effect of architecture. It was found that the macromolecular architecture can influence noticeably the micellar shape, size, and structure, depending on the relative crowding of arms in the core and the corona. 

The influence of macromolecular architecture on the micellar behavior of a series of miktoarm (polystyrene)_n_(polyisoprene)_n_, S_n_I_n_, star copolymers, comprising equal number *(n)* of polystyrene and polyisoprene arms, within a low molecular weight PI matrix has been previously investigated by our group [76]. The characteristics of the micelles were studied by small angle x-ray scattering, SAXS, as a function of *n*, with *n* between 1 and 16. The radius of the micellar core was found to be independent of the number of arms *n*, whereas the aggregation number decreased with increasing *n*, showing a *n*^−1^ power law dependence. In addition, the volume fraction of copolymer chains participating in the micelles was independent of *n*. The results showed that the junction point of these star copolymers did not significantly affect micellization. Those results were understood in terms of a simple thermodynamic model developed based on the Leibler approach; the predictions of theory are quantitatively consistent with the results of the experiment, validating the assumptions made within this simple model.

Herein, we extend the work on the influence of macromolecular architecture on micellization by investigating the formation of micelles when graft copolymers are introduced into a homopolymer matrix. A series of single graft (polyisoprene)_2_(polystyrene) (I_2_S) copolymers are blended with a low molecular weight polyisoprene homopolymer matrix, and the characteristics of the obtained micelles are investigated by SAXS as a function of the polystyrene volume fraction, *f_PS_*. All copolymers possess more-or-less the same overall molecular weight, while the copolymer concentration in the blends was kept constant at 2 wt%. The results for the graft copolymers are compared to those obtained for the corresponding linear polystyrene-*b*-polyisoprene (SI) copolymers and for a (polystyrene)_2_(polyisoprene) (S_2_I) graft copolymer, which is a mirror-image to one of the I_2_S graft copolymers. 

## 2. Materials and Methods

Materials: A series of (polyisoprene)_2_(polystyrene), I_2_S, 3-miktoarm star (graft) copolymers, and one (polystyrene)_2_(polyisoprene), S_2_I, graft copolymer were synthesized by anionic polymerization high-vacuum techniques and controlled chlorosilane chemistry approach. All intermediate and final products were rigorously characterized by size exclusion chromatography (SEC), membrane osmometry, low-angle laser light scattering (LALLS), and ^1^H-NMR spectroscopy. Details on the synthesis and characterization of these copolymers have been already reported in the literature [61,67,77,78,79,80,81]. For the sake of completeness, we briefly describe the synthetic procedure in the Supplementary Material. The three linear polystyrene-*b*-polyisoprene diblock copolymers, SI, that were studied here were synthesized by sequential anionic polymerization high-vacuum techniques. Benzene was the solvent, and *sec*-BuLi the initiator, with the styrene being polymerized first. Polyisoprene (PI) homopolymer was synthesized by anionic polymerization under Argon atmosphere. The authors acknowledge Dr. J. W. Mays and K. Hong for the synthesis and the kind donation of the diblock copolymers and the PI homopolymer. The molecular characteristics of samples are given in Table 1. All samples have approximately the same overall molecular weight and varying compositions. Note that in all I_2_S and SI samples, the polystyrene chain is perdeuterated (monomer: D8-styrene).

Sample Preparation: All the blends were prepared by dissolving the copolymer in HPLC grade tetrahydrofuran (THF), a common good solvent for both homopolymers. Weighted amounts of the low molecular weight polyisoprene to be used as the matrix were then added to the THF solution so as to obtain a 2 wt% mixture of the copolymers within the homopolymer, and the solutions were stirred for 24 h. THF was removed by slow evaporation at ambient temperature 20 °C, in a vacuum oven, under dynamic vacuum conditions. THF was left to evaporate for more than 48 h, and complete evaporation was verified by occasionally weighting the samples, until mass stabilization. This preparation method provides samples of well-dissolved copolymers within the low viscosity PI matrix. The samples were then placed inside capillary glass tubes of 2 mm diameter, appropriate for the SAXS measurements.

Small-angle X-ray Scattering: Small-angle X-ray scattering (SAXS) experiments were conducted at the Dutch–Belgian Beamline (DUBBLE, Grenoble, France) at the European Synchrotron Radiation Facility (ESRF) station BM26B [76,82]. SAXS data were recorded using a two-dimensional position-sensitive detector. The X-ray wavelength was 1.55 Å. Two different sample-to-detector distances were used, 3 and 7.5 m; thus, a wide scattering vector range was covered, 0.04 < *q* < 2 nm^−1^; the magnitude of the scattering vector is *q* = (4π/λ)sin θ, where 2θ is the scattering angle. All measurements were conducted at 25 °C.

The two-dimensional scattering images were radially averaged with respect to the center of the primary beam, and isotropic SAXS intensity profiles were obtained. The *q* scale was calibrated using a specimen of wet collagen (rat tail tendon) and Silver Behenate. Lupolen and Eltex were used as reference samples to calibrate the scattered intensity in absolute units (cm^−1^). The data have been normalized with respect to the intensity of the incident beam, in order to correct for primary beam intensity decay. Then, absorption, background scattering, and copolymer concentration corrections were applied. The incident and the transmitted beams were measured using two ionization chambers, placed before and after the sample. The background correction was made by subtracting from the total intensity the contribution of density fluctuations evaluated from measuring pure polyisoprene.

Data Analysis: The detailed description of the methodology followed for the analysis of the SAXS data is provided in the Supplementary Material and our previous related work [76]. In brief, the scattered intensity is analyzed considering a mono-disperse collection of particles, i.e.,

(1) $$I\left(q\right)=N_{p}V^{2}{\left(∆\rho \right)}^{2}P\left(q\right)S\left(q\right)=\Phi V{\left(∆\rho \right)}^{2}P\left(q\right)S\left(q\right)$$

where *N*_p_ is the number density of particles (namely, the micelles), *V* is the volume of the scattering particle, Φ is the volume fraction of the particles in the mixture, Δ*ρ* is the scattering length density contrast between the particles and their surroundings (solvent/matrix), *P*(*q*) is the particle form factor, and *S*(*q*) is the structure factor. The systems investigated herein are dilute; therefore, the structure factor can be neglected, i.e., *S*(*q*) = 1.

It is important to stress that the micelles in the present systems consist of a core formed mainly by the PS blocks of the copolymers and a corona formed by the PI blocks. PI chains of the matrix may penetrate in the corona. Therefore, the X-ray scattering length density contrast arises from the micellar cores, and the SAXS profiles represent the form factor of the cores. The form factor for a homogeneous sphere of radius *R* was sufficient to successfully analyze most data:

(2) $$P\left(q\right)=\frac{9{\left(sinqR-qRcosqR\right)}^{2}}{{\left(qR\right)}^{6}}$$

The distribution in core size was appropriately considered by applying a Gaussian distribution around the average core radius. The calculated intensity was fitted to the experimental data by adjusting *R* and the standard deviation of the distribution of *R* as well as a scaling factor related to the product *N*_p_(Δ*ρ*)^2^ (cf. Equation (1)).

The scattered intensity can also be used to evaluate the invariant *Q*, which describes the mean square fluctuations within the sample. The invariant was defined by Porod as $Q=\int_{0}^{\infty }I\left(q\right)q^{2}dq$. For an ideal two-phase system having sharp boundaries and constant densities within the phases, *Q* is equal to

(3) $$Q=2\pi^{2}\Phi \left(1-\Phi \right){\left(\Delta \rho \right)}^{2}$$

where Φ and 1−Φ are the volume fractions of the particles and the matrix. The forward scattered intensity is calculated as

(4) $$I_{0}=N_{p}V^{2}{\left(\Delta \rho \right)}^{2}=\Phi V{\left(\Delta \rho \right)}^{2}$$

These equations are used to calculate the number of scatterers *N*_p_, the contrast factor Δ*ρ*, the volume fraction of the scatterers Φ, the volume fraction of polystyrene in the micellar core *η_PS_*, as well as the copolymer volume fraction participating in micelles, ϕ_mic_, and that dissolved in the matrix as unimers, ϕ_uni_. All details are included in the Supplementary Material.

Finally, the aggregation number, e.g., the number of copolymer molecules participating in a micelle, can be estimated considering:

(5) $$Q_{m}=\frac{M_{core}}{M_{PSarm}}$$

where *M_core_* is the mass of the core and *M_PSarm_* is the mass of the polystyrene block in the copolymer. *M_core_* is derived from the volume *V* of the core and the mass density of polystyrene above T_g_ [83]. Since the forward scattering *I*_0_ is very sensitive to the scattering mass, the estimation of *M_core_* via the volume of the scatterer is considered to be more accurate than that based on the estimation of the core volume from the apparent radius of the core, which is derived from the form factor analysis.

## 3. Results and Discussion

**Figure 1:**
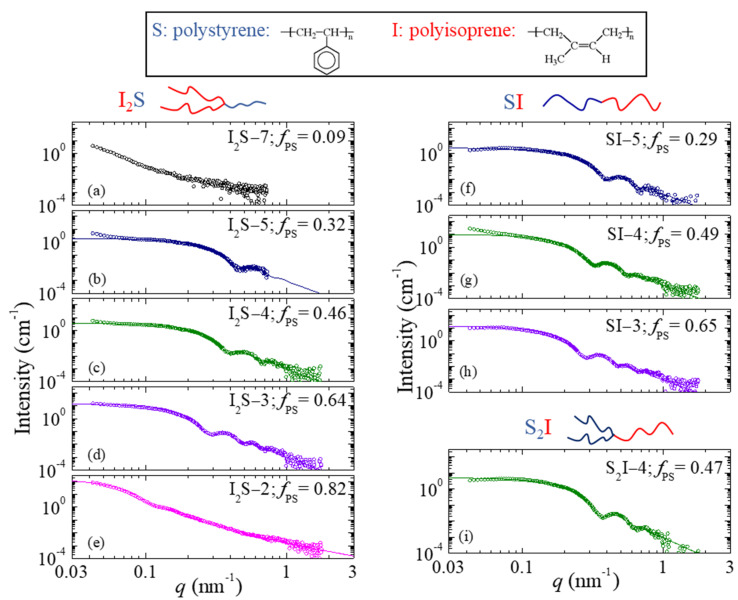
Small-angle x-ray scattering intensity profiles for the 2 wt% blends of (**a**–**e**) the various I_2_S graft copolymers and (**f**–**h**) the linear SI diblock copolymers and (**i**) the S_2_I−4 graft within a low molecular weight polyisoprene homopolymer matrix at room temperature. The corresponding best fits are shown by solid lines. Schematics that illustrate the chemical structures of all polymers studied in this work are included.

The scattering profiles measured for the 2 wt% blends of the I_2_S graft copolymers within the polyisoprene matrix for the various polystyrene compositions *f_PS_* are presented in Figure 1a–e. The profile corresponding to the graft copolymer I_2_S−7 (Figure 1a) with the lower polystyrene volume fraction is featureless, signifying that there are no micelles formed in that mixture. The slightly increasing intensity at low wavevectors can be attributed to electron density fluctuations due to polystyrene blocks that are dispersed within the polyisoprene matrix. It is noted that, for the I_2_S−7 copolymer, even the blend with 4 wt% copolymer did not exhibit scattering features that would indicate the existence of micelles; thus, it is deduced that the critical micelle concentration of the I_2_S−7 is higher than 4 wt%.

When the polystyrene content of the copolymer increases (Figure 1b–e), micellization takes place, and the scattering pattern exhibits the characteristic features of the form factor of spherical scatterers, namely a plateau in the low *q* regime and oscillations with well-pronounced minima, the position of which agrees with the condition fulfilled by the minima corresponding to the form factor of a homogeneous sphere (*qR* = 4.493, 7.725, etc.). As the composition *f_PS_* increases from 0.32 to 0.64, the position of the first minimum shifts towards lower *q* values, implying that the radius of the micellar core, *R_c_*, increases. In the case of the sample with *f_PS_* = 0.82, the plateau region is not evident in the accessed *q* range; however, two-week minima can be observed, at even lower wavevectors with respect to the previous copolymers, and the curve can still be fitted very well with the form factor of a homogeneous sphere. Finally, the scattering profile of the blend containing the graft copolymer with the highest polystyrene volume fraction (*f_PS_* = 0.90) is rather complex and does not correspond to the scattering from spherical particles. This sample does not form micelles but tends to phase separate; it will be discussed separately at the end of this section. 

Figure 1f–h present the scattering profiles obtained from the 2 wt% blends of PI with the three linear SI diblock copolymers, whereas Figure 1i is that of the blend with the S_2_I−4 graft copolymer, which is the mirror image of I_2_S−4. All of them exhibit the characteristic features of the form factor of a homogeneous sphere with at least two well observed minima. Similarly to the case of the graft copolymers, the position of the first minimum of the curve shifts to lower wavevectors as the polystyrene volume fraction in the copolymers increases.

**Figure 2:**
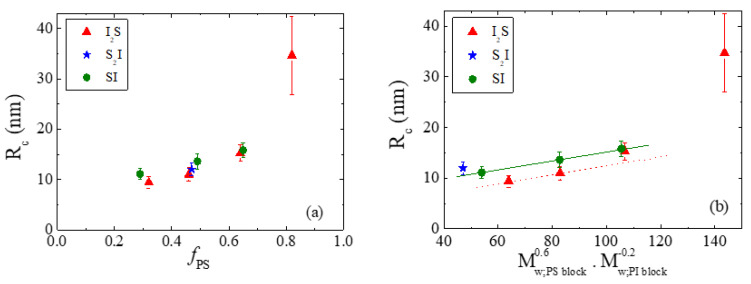
(**a**) The composition dependence of the radius *R_c_* of the micellar core formed in the 2 wt% blends of the I_2_S graft (solid triangles), the S_2_I graft (star), and the linear copolymers (solid circles), within the polyisoprene matrix at room temperature, as derived from the form factor analysis. The error bars correspond to the fitted polydispersity. (**b**) Core radius of the micelles formed in the 2 wt% blends of the I_2_S (solid triangles) and S_2_I (star) grafts, and the SI diblock copolymers (solid circles) in the polyisoprene matrix at room temperature, as a function of the particular relations to the molecular weights of the core-forming (PS) and corona-forming (PI) blocks (see text). The solid line corresponds to the data for the SI diblocks, whereas the dotted line has the same slope with the solid one and is followed by the I_2_S data only at low values of *f_PS_*.

The fitting results for the micellar core radius are presented in Figure 2a as a function of the copolymers composition, *f_PS_*. The overall molecular weight of all copolymers under study has been kept almost constant, so that *f_PS_* and the macromolecular architecture are the only variables. The error bars refer to the polydispersity in radius, calculated from the form factor analysis, which, in most cases, is around 10% of the radius. For both the linear and the graft copolymer micelles, the core radius increases with increasing *f_PS_*. This is in agreement with previous studies on linear copolymer micelles, where the radius of the micellar core was reported to depend strongly on the molecular weight of the core-forming block [49,53]. A closer look at the data suggests that the core size of the micelles formed by the graft copolymers increases more rapidly than that of the micelles formed by the linear copolymers, signifying that the polystyrene blocks in the core tend to be more stretched. Given the similar molecular weight of the polystyrene blocks in the graft copolymers and in the corresponding linear ones, this difference could either originate from the grafting of the polyisoprene corona-forming block with the second arm or from the halving of the length of the polyisoprene block. The dependence of the core radius on the molecular weights of the core- and the corona-forming blocks has been reported in the literature [49,53], and the empirical scaling laws $R_{c}\propto M_{w,core\_block}^{0.6}$ and $R_{c}\propto M_{w,corona\_block}^{-0.2}$ have been proposed [53]. To dissociate the effects of the block molecular weights and the architecture (i.e., grafting), Figure 2b shows the core radius as a function of the product of these two dependencies. For the linear copolymer micelles, the core radii obey a linear relation on the product $M_{w,core\_block}^{0.6}M_{w,corona\_block}^{-0.2}$, signifying that, for constant homopolymer molecular weight, the micellar size depends solely on the lengths of the two blocks. On the contrary, the core radii of the graft copolymer micelles display a non-linear behavior on the product, implying that the increase in polystyrene molecular weight and the decrease in polyisoprene block length are not enough to account for the behavior of these micelles. Therefore, architecture and, in particular, grafting the second corona-forming block on the copolymer drive the enhanced stretching of the polystyrene blocks and, consequently, the increased core size.

**Figure 3:**
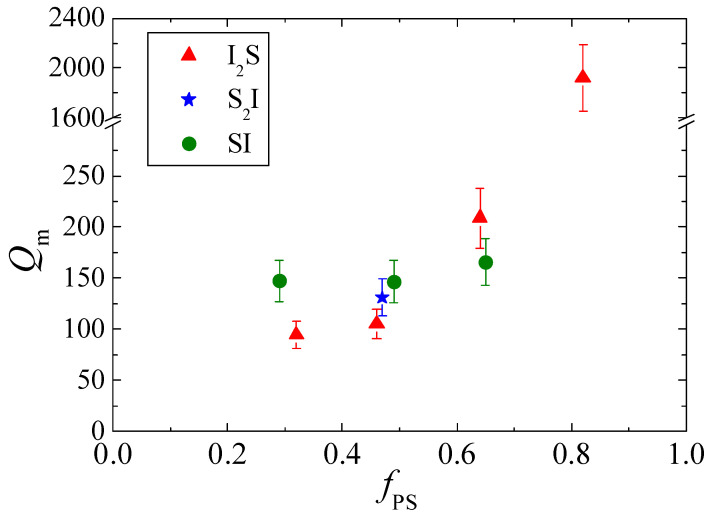
The aggregation number *Q_m_* of the micelles, calculated from the analysis of the forward scattering, for the 2 wt% blends of the I_2_S graft (solid triangles), the S_2_I graft (star), and the linear copolymers (solid circles) within the polyisoprene matrix at room temperature, as a function of the polystyrene volume fraction of the copolymer.

The aggregation number *Q_m_* can provide complementary information on the micellar structure. As shown in Figure 3, the aggregation number of the graft copolymer micelles increases rapidly with increasing *f_PS_*, while a much weaker dependence is observed in the case of the linear copolymer micelles. Qualitatively, this finding is consistent with the increase in core radius found for both series of copolymer micelles. The faster increase obtained for the graft copolymer micelles should be related to the increased stretching discussed previously. Note that due to stretching, more chains can be accommodated in the same core volume. Alternatively, this more abrupt increase of *Q_m_* derived for the graft copolymer micelles could be related to the penetration of homopolymer chains within the core. In this scenario, the number of copolymer chains needed to form a micelle with the same core radius would decrease. We calculated the Flory–Huggins phase diagram of the corresponding polystyrene/polyisoprene blend. The results show that immiscibility does not allow for the incorporation of a significant number of polyisoprene matrix chains within the core, for the range of PS molecular weights studied here. Therefore, this scenario should be excluded. Moreover, the ratio of the geometrical volume of the scatterers (calculated based on the core radius) to the volume of the scatterers estimated by the form factor analysis (derived by *I*_0_, equation (4), and in the Supplementary Material) can provide an indication about the polystyrene volume fraction within the core. For the micelles understudy, this ratio is found to have a constant value, independent of both the molecular weight and the copolymer architecture, reinforcing the validity of the results. Thus, the faster increase in the aggregation number with composition obtained for the micelles of the graft copolymers compared to those of the linear ones can only be attributed to better chain packing due to the enhanced stretching of the polystyrene chains that form the micellar core. Once more, the origin of this behavior is related to the specific architecture of the graft copolymers.

The volume fraction of the copolymer chains that participate in micelles ϕ_mic_, and of the free unimers that remain dispersed in the polyisoprene matrix, ϕ_uni_, have been estimated for the systems under study. It was found that ϕ_mic_ is more or less constant, independent of the copolymers molecular characteristics or their architecture, equal to around 60% of the total copolymer volume fraction in the blends. This implies that the number density of micelles in the blends *N*_p_ decreases as the copolymers’ composition *f_PS_* increases, in order to compensate for the increase in core radius. Nonetheless, for the graft copolymer with *f_PS_* = 0.82, ϕ_mic_ decreases to ~30% of the total copolymer volume fraction. This low value could be attributed to an incipient macrophase separation in that system, which is driven by the high molecular weight of the polystyrene block. Macrophase separation is not evident in the SAXS data, whereas the fact that this sample is transparent by eye, similar to the samples of lower *f_PS_*, makes this scenario less probable. Yet, our data and observations are not conclusive.

Interesting information on the effect of macromolecular architecture on micelles characteristics is obtained by comparing the results for the I_2_S−4 and S_2_I−4 mirror-like graft copolymers and their corresponding linear SI−4 diblock. These three copolymers have almost the same total molecular weight (we acknowledge that the molecular weight of SI−4 is by ~20% slightly higher) and composition (*f_PS_* ≈ 0.5), but differ in the number of polyisoprene versus polystyrene arms. The molecular characteristics of the copolymers are presented again in Table 2, along with the experimental results concerning the core radius and the aggregation number. The data show that the core radius increases in the order I_2_S−4 < S_2_I−4 < SI−4. Although the molecular weight of the polystyrene core-forming block of S_2_I−4 is about half that of I_2_S−4, its core radius is, interestingly, slightly larger than that of I_2_S. This is inconsistent to the $R_{c}\propto M_{w,core\_block}^{0.6}$ power law that has been derived for micelles formed by linear copolymers [53]. Since the phase diagram of the polystyrene/polyisoprene blend does not support the scenario of mixing polyisoprene homopolymer chains within the micellar core, the unexpectedly large core radius of S_2_I−4 is a result of the specific architecture of this copolymer and should be related to the stretching of the core-forming blocks. Note that stretching in the case of S_2_I is much more pronounced than in the case of I_2_S, showing that the nature of the grafted block (i.e., core-forming or corona-forming) and, consequently, its position in the micelle, has a significant impact on micellization. Consistently to what has been observed for the S_n_I_n_ miktoarm copolymer micelles [76], the number of blocks per copolymer junction point does not play an important role in the micellization of Y-shaped graft copolymers; however, the spatial asymmetry induced by the placement of the graft block in the core or the corona has a significant impact on the micelle characteristics.

Similar to what has been observed in the case of I_2_S, one could expect a high aggregation number for S_2_I−4, driven by the better packing of the stretched chains within the core. Indeed, our data show that the aggregation number of S_2_I−4 is bigger than that of I_2_S−4, and increases in the order I_2_S−4 < S_2_I−4 < SI−4, following the trend observed for the core radius. Taking into account that, for S_2_I, there are two polystyrene arms per molecule, it follows that the number of polystyrene blocks per micellar core is almost triple in the case of S_2_I−4 with respect to I_2_S−4. This implies that the low molecular weight of the polystyrene blocks in S_2_I−4 is counterbalanced by their high number per micellar core, resulting in micelles of similar sizes. These findings suggest that the size of the micellar core is not only driven by the length of the core-forming blocks, but mostly by a critical volume necessary for the formation of stable micelles. Note that similar trends for the core radius and the aggregation number have been reported for the micellization of the same copolymers in a selective solvent [61].

Regarding the copolymers with high *f_PS_*, where architectural asymmetry becomes important due to the much shorter length of the polyisoprene blocks with respect to that of polystyrene, one may expect that micellization could result in micelles of non-spherical shapes. Up to *f_PS_* = 0.82, our SAXS data show that spherical micelles are formed, disregarding the incipient macrophase separation alluded for the I_2_S−2 blend based on its low ϕ_mic_. Interestingly, the phase behavior is significantly altered for the 2 wt% blend of the graft copolymer with *f_PS_* = 0.90.

**Figure 4:**
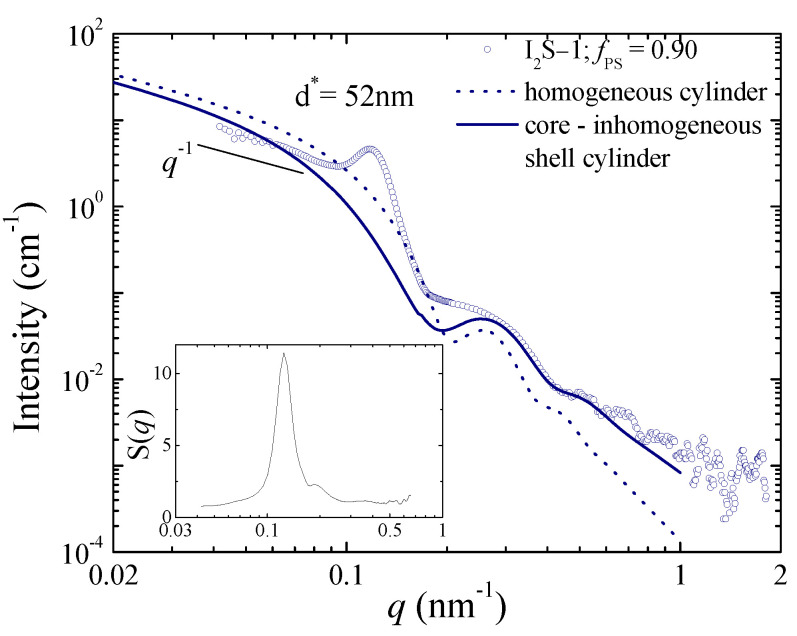
The scattering profile for the 2 wt% blend of the I_2_S−1 graft copolymer within the polyisoprene matrix at room temperature. The simulated scattering curves for homogeneous cylinders with *R* = 19.8 nm (dotted line) and for core-shell cylinders with inhomogeneous shell and an inner radius of 22 nm and an outer one of 30 nm (solid line) are included as well. The inset shows the structure factor estimated using the scattering data and the form factor of the core-inhomogeneous shell cylinders.

As it was already mentioned, the scattering profile of this blend is rather complex and does not correspond to the form factor of spherical scatterers. Certain strong features are present in its scattering pattern (Figure 4) that could allow a rough estimate of its structural characteristics. A broad but strong peak is observed around 0.12 nm^−1^, which is attributed to interparticle interference scattering. This corresponds to a characteristic mean distance between the scatterers of approximately 52 nm. No higher-order peaks are evident, suggesting that this lattice is quite disordered. Moreover, a *q*^−1^ dependence is apparent in the low *q* range, which is characteristic of the scattering from rod-like particles, whereas a clear minimum appears around 0.4 nm^−1^, possibly followed by higher-order minima. Thus, we can safely derive that there is an interacting system of elongated particles with a spacing of around 52 nm. In order to have a rough estimate of the characteristics of the particles, various form factors that describe elongated particles were employed to simulate the experimental curve (neglecting for the moment the interference peak). The simulated curve that corresponds to scattering from homogeneous cylinders of 19.8 nm radius is included in Figure 4 (dotted line). The *q*^−1^ dependence and the minimum at 0.4 nm^−1^ are both reproduced, yet the intensity does not comply well with the experimental values, which predisposes toward a core-shell structure. Indeed, the agreement is improved when the form factor of core–homogeneous shell cylinders is used. Yet, the best conformity is obtained for the form factor of core–inhomogeneous shell cylinders [84]. An algebraic density profile has been used to describe the inhomogeneous shell, which has been successfully employed in the past to describe systems with grafted polymer chains [84] like those under study. It should be kept in mind that, in reality, the soluble block of the copolymer forms a polymer brush that surrounds the insoluble core. The simulated curve that best describes the form factor contribution to the experimental data is included in Figure 4 (solid line) and corresponds to cylindrical particles with an inner radius of 22 nm and an outer radius of 30 nm. Using this form factor, the structure factor contribution is dissociated and presented in the inset of Figure 4. It exhibits a relatively strong peak with a full width at half maximum that would correspond to a coherence length of 161 nm or three times the characteristic distance of 52 nm.

An interesting finding is that the electron density of the core is lower than that of the shell, implying that the polyisoprene block resides in the core while the polystyrene blocks form the shell, unlike what has been observed and discussed for the other systems. This points to a macrophase separation between copolymer-rich domains and homopolymer-rich regions having occurred. The fact that this sample is not transparent, in contrast to all other specimens, supports this scenario. Macrophase separation is driven by the high molecular weight of the polystyrene arm with respect to the polyisoprene blocks that promotes immiscibility with the homopolymer matrix. The copolymer chains that form the copolymer rich domains do not feel the polyisoprene homopolymer chains and, thus, adopt the morphology expected for the bulk state, with the minority component (namely, the polyisoprene arms) forming the core of cylinders, which are dispersed within the polystyrene (blocks) matrix. This morphology is in agreement with that reported for the same copolymer, both in its pure (bulk) state [67] and blended with small amounts of low molecular weight polyisoprene [80]. It was found that the pure copolymer forms hexagonally packed polyisoprene cylinders in a polystyrene matrix with a lattice spacing of 31 nm [67], while bilayered sheets and closed vesicles were observed upon blending [80]. Using simple geometry and applying the space-filling condition for the pure copolymer case, one could derive the radius of the polyisoprene cylinders as *R*_PI_ = 6.2 nm and the distance between the cylinders as *d*_PS_ = 23 nm. One would expect that the same core radius should more-or-less apply in the 2 wt% blend under study. However, the larger radius we derive from the SAXS analysis implies that polyisoprene homopolymer resides within the cylinder core. Assuming hexagonal packing of the cylinders and considering that the distance between them is equal to that calculated for the pure copolymer, the radius of the PI core can be estimated to be around 18 nm, which is very close to the experimentally obtained value.

## 4. Conclusions

The micellization of single graft copolymers within a homopolymer matrix has been investigated. Our data show that spherical micelles are formed at intermediate volume fractions of the graft copolymer’s core-forming block, 0.32 ≤ *f_PS_* ≤ 0.82. At lower *f_PS_*, the copolymer is miscible with the polyisoprene homopolymer matrix, whereas macrophase separation takes place for *f_PS_* = 0.90. In this latter case, copolymer-rich domains are embedded within the homopolymer matrix. These domains consist of cylinders formed by the polyisoprene blocks swollen with polyisoprene homopolymer, dispersed in a matrix of the polystyrene blocks.

When the graft copolymers form micelles, the core radius increases with the increasing volume fraction of the core-forming block, *f_PS_*. This increase is more pronounced for the micelles formed by graft copolymers than for the ones formed by the respective linear copolymers, demonstrating that the core-forming blocks are more stretched in the case of graft copolymers. We have shown that this extra stretching is a purely architectural effect that stems from grafting the second corona-forming block. Higher stretching allows the chains to better pack within the core, and, thus, higher aggregation numbers are obtained for the graft copolymer micelles than for the ones formed by the linear diblocks. Grafting a second core-forming block instead of a second corona-forming one (i.e., S_2_I versus I_2_S) results in even more remarkable changes. The core radius does not depend as strongly on the molecular weight of the core-forming block as is the case for the linear and I_2_S copolymers. The high aggregation number of the S_2_I micelles counterbalances the low molecular weight of their core-forming block, indicative of the fact that a critical core volume may be necessary for the formation of stable micelles, rather than a critical molecular weight of the core-forming blocks. Moreover, the comparison between the S_2_I, I_2_S, and SI micelles shows that the number of arms attached on the copolymer junction point is a less important architectural parameter when compared to the ratio of core-forming versus corona-forming arms.

Synthesizing complex macromolecular architectures is surely appealing and challenging from a chemistry point of view. Our results show that complex architectures can be also technologically appealing, since macromolecular architecture is proved to be a parameter that impacts micellar characteristics. As a next step, its impact on the critical micellization concentration (CMC) should be explored, given that for an efficient use of copolymers as compatibilizers in immiscible blends, high values of the CMC are preferred.

## Figures and Tables

**Table 1:** Molecular characteristics of (polyisoprene)_2_(polystyrene), (polyisoprene)(polystyrene)_2_ single graft copolymers, and polyisoprene-b-polystyrene linear copolymers.

Species	*M_w_* ^a^	*M_w_* ^b^	Ɖ = *M_w_*/*M_n_* ^b^	*w*_PS_^c^	*N* ^d^	*f_PS_* ^e^
I_2_S−1	106,000	94,100	1.04	0.91	1191	0.90
I_2_S−2	90,800	83,700	1.04	0.84	1031	0.82
I_2_S−3	87,400	88,100	1.04	0.67	1017	0.64
I_2_S−4	92,000	101,200	1.04	0.49	1098	0.46
I_2_S−5	89,800	104,300	1.04	0.35	1093	0.32
I_2_S−7	91,300	118,400	1.06	0.10	1149	0.09
S_2_I−4	93,000	102,500	1.06	0.48	1112	0.47
SI−3	116,300	138,600	1.02	0.68	1354	0.65
SI−4	113,700	146,400	1.05	0.52	1351	0.49
SI−5	99,500	140,100	1.03	0.32	1213	0.29
PI	-	4000	1.06	0.00	81	0.00

^a^: weight-average molecular weight in g/mol measured by LALLS in THF at 25 °C; ^b^: weight-average molecular weight in g/mol and polydispersity index by size exclusion chromatography in THF at 25 °C, utilizing polystyrene standards (UV detector). RI detector for PI homopolymer; ^c^: polystyrene weight fraction by ^1^H-NMR or SEC-UV using polystyrene area calibration at 260 nm; ^d^: number of segments of the copolymers or homopolymer based on average segmental volume; ^e^: polystyrene volume fraction.

**Table 2:** The molecular characteristics and the SAXS results for the mirror-like I_2_S and S_2_I copolymers, as well as their linear counterpart, SI−4.

Species	*M_w,PS block_*	*M_w,PI block_*	*R_c_* (nm)	*Q_m_*	*Q_m_*×(PS arms)
I_2_S−4	45,080	23,460	11.0	105	105
S_2_I−4	22,320	48,360	12.0	131	262
SI−4	59,120	54,580	13.6	147	147

## Data Availability

The data included in this report are available upon request to the corresponding authors.

## Supplementary Materials

The following are available online at https://www.mdpi.com/2073-4360/13/5/833/s1. Materials Synthesis and SAXS data analysis.
