# Supplementary material for: The Micellization of Well-Defined Single Graft Copolymers in Block Copolymer/Homopolymer Blends
Source: Polymers (Basel). 2021 Mar 9;13(5):833. doi: 10.3390/polym13050833 (PMC7967213; doi:10.3390/polym13050833)
Supplement: Supplementary file 1 [file polymers-13-00833-s001.pdf]

Supplementary Material

The Micellization of Well-Defined Single Graft Copolymers in Block Copolymer/Homopolymer Blends

Eleni Pavlopoulou,[1*] Kiriaki Chrissopoulou,[1] Stergios Pispas,[2,3] Nikos Hadjichristidis[3,4] and Spiros H. Anastasiadis[1,5*]

[1] Institute of Electronic Structure and Laser, Foundation for Research and Technology - Hellas, P. O. Box 1527, 711 10 Heraklion Crete, Greece
[2] Theoretical and Physical Chemistry Institute, National Hellenic Research Foundation, 48 Vassileos Constantinou Ave., 11635 Athens, Greece
[3] Department of Chemistry, University of Athens, 157 71 Zografou, Athens, Greece
[4] Polymer Synthesis Laboratory, Physical Sciences and Engineering Division, King Abdullah University of Science and Technology (KAUST), Thuwal 23955, Saudi Arabia
[5] Department of Chemistry, University of Crete, 710 03 Heraklion Crete, Greece

* Correspondence: epavlopoulou@iesl.forth.gr, spiros@iesl.forth.gr

Materials:

Three linear polystyrene-*b*-polyisoprene diblock copolymers, SI, were synthesized by sequential anionic polymerization high-vacuum techniques, starting from styrene, at room temperature in benzene with *sec*-BuLi as the initiator. The polyisoprene (PI) homopolymer matrix was synthesized by anionic polymerization under Argon atmosphere in benzene with *sec*-BuLi. The authors acknowledge Dr. J. W. Mays and K. Hong for the synthesis and the kind donation of these polymers. The molecular characteristics of the samples are given in Table 1. All samples have approximately the same overall molecular weight and varying compositions.

A series of (polyisoprene)$_2$(polystyrene), I_2S, 3-miktoarm star copolymers (single grafts) were synthesized, using high-vacuum techniques in glass reactors provided with break-seals for addition of reagents and constrictions for removal of products, by anionic polymerization and controlled chlorosilane chemistry. Details on the synthesis and characterization of these systems have been described earlier [1-7]. The monomers (styrene and isoprene), the solvent (benzene) and the linking agent (methyltrichlorosilane) were purified to the standards required for anionic polymerization high-vacuum techniques, following well-known procedures [2,3,7]. Living polystyrenyl-lithium (PSLi) and polyisoprenyl-lithium (PILi) precursors were synthesized in benzene using *sec*-butyllithium as initiator. A ~3% w/v solution of PSLi in benzene was allowed to react with a large excess (SiCl/Li = 100) of methyltrichlorosilane (CH_3SiCl_3) to ensure the production of PS-Si(CH_3)Cl_2, followed by removal of the unreacted CH_3SiCl_3 and benzene on the vacuum line. Next, an excess of PILi in benzene was added to a benzene solution of the macromolecular difunctional linking agent, PS-Si(CH_3)Cl_2, to obtain the CH_3Si(PS)(PI)$_2$ 3-miktoarm star copolymers I_2S. The

excess PILi was deactivated with degassed methanol. The same procedure was applied to the synthesis of the (polystyrene)$_2$(polyisoprene), S$_2$I, 3-miktoarm star copolymer where the first step involved the incorporation of polyisoprene arm (adding PILi) and the second the incorporation of the two polystyrene arms (adding excess of PSLi). The desired I$_2$S or S$_2$I graft was isolated from the reaction mixture by solvent/nonsolvent fractional precipitation.

The fractionated final, as well as the intermediate products, were rigorously characterized by size exclusion chromatography (SEC) with both RI and UV detectors, membrane osmometry and low-angle laser light scattering (LALLS). Note that in all I$_2$S and SI samples, the polystyrene sequence is perdeuterated (monomer: D8-styrene). Therefore, the weight fraction was calculated by SEC-UV using the area under the elution peak for each star and the known concentration of copolymer injected, recorded at 260nm (the characteristic absorption band for phenyl rings). The area corresponds directly to the amount of styrene in the copolymer since isoprene segments do not absorb in this spectral region. A calibration curve was constructed for this reason by SEC-UV using a narrow polystyrene standard, injected at different known concentrations and recording the area of the elution peak, observed at 260nm in each case.

^{1}H-NMR was utilized for determining the microstructure of polyisoprene arms in the stars and the composition of the linear diblock copolymers. A typical ^{1}H-NMR spectrum is shown below for the diblock copolymer SI-5 (Fig. S1). For this purpose the peaks at 6.4-7.2 ppm (c) assigned to aromatic protons of the phenyl rings of styrene (5H), the peak at ca. 5.1 ppm (a) for 1,4-microstructure of polyisoprene (vinyl, 1H), and the peaks at ca. 4.8 ppm (b) for 3,4-microstructure of polyisoprene (vinyl, 2H), were utilized.

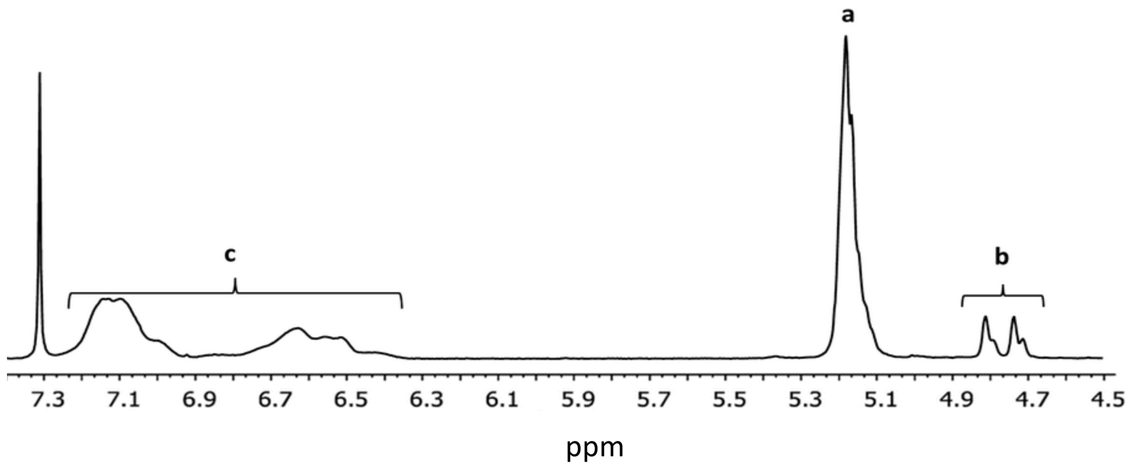

Figure S1. ^{1}H-NMR spectrum for the diblock copolymer SI-5.

Data Analysis:

For a mono-disperse collection of particles the scattered intensity can be written as

$$I(q) = N_p V^2 (\Delta\rho)^2 P(q) S(q) = \Phi V (\Delta\rho)^2 P(q) S(q) \quad (1)$$

where N_P is the number density of particles (micelles), V is the volume of the scattering particle, Φ is the volume fraction of the particles in the mixture, $\Delta\rho$ is the difference in scattering length density between the

particles and the solvent/matrix, $P(q)$ is the particle form factor, and $S(q)$ is the structure factor. For dilute systems, like the ones of the present study, the structure factor can be neglected, $S(q) = 1$.

The micelles in the present systems consist of a core formed mainly by the PS block of the copolymers and a surrounding corona formed by the PI blocks, swollen or not by the PI chains of the matrix. Thus, the X-ray scattering arises solely from the micellar cores; the SAXS data can be analyzed to provide the form factor of the cores. For all data presented in this work, the assumption of spherical micelles was sufficient to analyze the data. Thus, the form factor for a homogeneous sphere of radius R was used:

$$P(q) = \frac{9(sinqR - qRcosqR)^2}{(qR)^6} \tag{2}$$

Inevitably, there is some distribution in the size of the micelles as well as a finite interface region between the core and the corona. The polydispersity in the micellar core radius can be accounted for by writing

$$I(q) = N_p(\Delta\rho)^2 \int_0^\infty \left(V(r)\right)^2 P(q,r) f(r) dr \tag{3}$$

where $f(r)$ is the Gaussian distribution around the average core radius R

$$f(r) = \frac{1}{\sqrt{2\pi}\sigma} \exp\left(-\frac{(r-R)^2}{2\sigma^2}\right) \tag{4}$$

with σ the standard deviation of the distribution.

The calculated intensity was fitted to the experimental data by adjusting the two parameters R and σ as well as a scaling factor related to the product $N_P(\Delta\rho)^2$. The fitting was accomplished using a nonlinear least squares fitting procedure. The error or uncertainty associated with the determination of each of the fitting parameters can be determined by a manual iterative fitting procedure, by varying one parameter while keeping the others fixed and determining the necessary deviation to cause a notable divergence of the predicted scattering from the experimental data.

The scattered intensity can also be used to evaluate the invariant Q, which describes the mean square fluctuations within the sample. The invariant was defined by Porod as

$$Q = \int_0^\infty I(q) q^2 dq \tag{5}$$

For an ideal two-phase system having sharp boundaries and constant densities within the phases, Q is equal to

$$Q = 2\pi^2 \Phi(1-\Phi)(\Delta\rho)^2 \tag{6}$$

where Φ and 1-Φ are the volume fractions of the two phases. The forward scattering intensity is given by

$$I_0 = \Phi V (\Delta\rho)^2 \tag{7}$$

Thus, the volume fraction of the scattering particles, Φ, and the volume of the scatterer, V, can be calculated for a given excess scattering length density, providing a second, independent way for the determination of the radius R.

In general, the micellar core could be considered as a homogeneous particle formed by the polystyrene blocks of the copolymers and a finite fraction of polyisoprene homopolymer chains that may penetrate within the core. By denoting with η_{PS} the volume fraction of styrene in the core, the contrast between the core particle and the surrounding medium (matrix) becomes

$$\Delta\rho = \eta_{PS} \Delta\rho_{SI} \tag{8}$$

where $\Delta\rho_{SI}$ is the excess electron density between pure polystyrene and pure polyisoprene, $\Delta\rho_{SI} = \rho_{PS} - \rho_{PI}$.

The volume fraction ϕ of the total styrene units in the blend can be written as the sum of the styrene participating in micelles, ϕ_{mic}, and that dissolved in the matrix as unimers, ϕ_{uni}, i.e.,

$$\phi = \phi_{\mathrm{mic}} + \phi_{uni} = \eta_{PS}\Phi + \phi_{uni} \qquad (9)$$

In order to proceed with the solution of the set of equations 6 – 9, one has to make an assumption about one of the four unknown parameters. Roe [8] related the unimer volume fraction to the critical micellization concentration, $\phi_{uni}\approx\phi_{CMC}$, and considered it to be negligible with respect to ϕ_{mic} ($\phi_{uni}<<\phi_{mic}$), resulting in very low values of η_{PS} as the micelles dissolve with temperature. On the other hand, Gohr, et al. [9] assumed that the micellar core consists solely of polystyrene and estimated the unimer volume fraction to be comparable to ϕ_{mic}. Due to the strong segregation between polystyrene and polyisoprene, one can anticipate that, for the present systems, the volume fraction of polystyrene in the core should be very close to one. The same assumption was made in an earlier work of ours [10], where it was found that the results of the analysis were in very good agreement with theoretical predictions. Under this assumption, one can calculate the volume V of the micellar core, the core volume fraction in the blends, Φ, and the unimer volume fraction, ϕ_{uni}. In the following discussion, the volume fractions $\Phi = \phi_{mic}$ and ϕ_{uni} will be referring to the volume fractions of the copolymer chains participating in micelles and the free copolymer chains, respectively, keeping the same notation.

The aggregation number, e.g., the number of copolymer molecules participating in a micelle, can be estimated as well using the equation

$$Q_m = \frac{M_{core}}{M_{PSarm}} \qquad (10)$$

where M_{core} is the mass of the core and $M_{PS\,arm}$ is the mass of the polystyrene block in the copolymer. The mass M_{core} is derived from the volume V of the core and the mass density of polystyrene above T_g [11]. Since the forward scattering I_0 is very sensitive to the scattering mass, the estimation of M_{core} via the volume of the scatterer is considered to be more accurate than that based on the estimation of the core volume from the apparent radius of the core, which is derived from the form factor analysis. Finally, the number density N_P of micelles in the solutions can be estimated by the equation $\Phi = N_P V$.

References:

1. Pochan, D.J.; Gido, S.P.; Pispas, S.; Mays, J.W.; Ryan, A.J.; Fairclough, J.P.A.; Hamley, I.W.; Terrill, N.J. Morphologies of microphase-separated A(2)B simple graft copolymers. *Macromolecules* **1996**, *29*, 5091-5098.
2. Iatrou, H.; Hadjichristidis, N. Synthesis of a Model 3-Miktoarm Star Terpolymer. *Macromolecules* **1992**, *25*, 4649-4651.
3. Iatrou, H.; Siakalikioulafa, E.; Hadjichristidis, N.; Roovers, J.; Mays, J. Hydrodynamic Properties of Model 3-Miktoarm Star Copolymers. *Journal of Polymer Science Part B-Polymer Physics* **1995**, *33*, 1925-1932.
4. Pochan, D.J.; Gido, S.P.; Pispas, S.; Mays, J.W. Morphological transitions in an I2S simple graft block copolymer: From folded sheets to folded lace to randomly oriented worms at equilibrium. *Macromolecules* **1996**, *29*, 5099-5105.
5. Yang, L.Z.; Gido, S.P.; Mays, J.W.; Pispas, S.; Hadjichristidis, N. Phase behavior of I2S single graft block copolymer/homopolymer blends. *Macromolecules* **2001**, *34*, 4235-4243.
6. Anastasiadis, S.H.; Chrissopoulou, K.; Fytas, G.; Fleischer, G.; Pispas, S.; Pitsikalis, M.; Mays, J.W.; Hadjichristidis, N. Self-diffusivity in block copolymer solutions .2. A(2)B simple grafts. *Macromolecules* **1997**, *30*, 2445-2453.

7. Pispas, S.; Hadjichristidis, N.; Potemkin, I.; Khokhlov, A. Effect of architecture on the micellization properties of block copolymers: A(2)B miktoarm stars vs AB diblocks. *Macromolecules* **2000**, *33*, 1741-1746.
8. Rigby, D.; Roe, R.J. Small-Angle X-Ray-Scattering Study of Micelle Formation in Mixtures of Butadiene Homopolymer and Styrene Butadiene Block Copolymer. *Macromolecules* **1984**, *17*, 1778-1785.
9. Gohr, K.; Schartl, W.; Willner, L.; Pyckhout-Hintzen, W. SANS investigation of PS-PB block copolymer micelles in a short chain PB homopolymer matrix. *Macromolecules* **2002**, *35*, 9110-9116.
10. Pavlopoulou, E.; Anastasiadis, S.H.; Iatrou, H.; Moshakou, M.; Hadjichristidis, N.; Portale, G.; Bras, W. The Micellization of Miktoarm Stan S_nI_n Copolymers in Block Copolymer / Homopolymer Blends. *Macromolecules* **2009**, *42*, 5285-5295.
11. Richardson, M.J.; Savill, N.G. Volumetric Properties of Polystyrene - Influence of Temperature, Molecular-Weight and Thermal-Treatment. *Polymer* **1977**, *18*, 3-9.
